# New Insight on Hydrogen Evolution Reaction Activity of MoP_2_ from Theoretical Perspective

**DOI:** 10.3390/nano9091270

**Published:** 2019-09-05

**Authors:** Yuyue Gao, Hongyan Li, Jingyu Wang, Jianyi Ma, Haisheng Ren

**Affiliations:** 1 Institute of Atomic and Molecular Physics, Sichuan University, Chengdu610065, China; 2 School of Chemical Engineering, Sichuan University, Chengdu 610065, China; 3 School of Aeronautics and Astronautics, Sichuan University, Chengdu 610065, China; wangjingyu@scu.edu.cn

**Keywords:** hydrogen evolution reaction, periodic density functional theory, hydrogen coverage, catalytic performance, MoP_2_

## Abstract

We systematically investigated the hydrogen evolution reaction (HER) of six facets of ${\mathrm{MoP}}_{2}$ based on the periodic density functional theory (DFT). The calculated values of Gibbs free energy of hydrogen adsorption ($\Delta G_{H}$) indicated that the (111) facet has a good HER activity for a large range of hydrogen coverages. The zigzagged patterns before 75% hydrogen coverage suggest a facilitation among Mo1, P1 and Mo2 sites, which are attributed to repeat occupancy sites of H atoms. From ab initial atomistic thermodynamics analysis of hydrogen coverage, we gained that the most stable coverage of hydrogen is 18.75% at 1 atm $H_{2}$ and 298 K. Finally, the doping effects on HER activity were investigated and found that catalytic performance can be improved by substituting P with an S or N atom, as well as substituting the Mo atom with an Fe atom, respectively. We hope this work can provide new insights on further understanding of HER for ${\mathrm{MoP}}_{2}$ and give instructions for the experimental design and synthesis of transition metal phosphides (TMPs)-based high-performance catalysts.

## 1. Introduction

Hydrogen has been regarded as a promising energy carrier and future sustainable high energy fuel, due to its properties of non-pollution, energy conversion and gravimetric energy density [1,2]. The energetic efficiency, environmental sustainability and carbon-free alterative of hydrogen generations come from the interconversion between water and hydrogen. Electrolytic water-splitting [3] $\;(H_{2}O\left(l\right)\to H_{2\;}\left(g\right)+\frac{1}{2}O_{2}\left(g)\right)$ is one of the most reliable and effective methods to produce hydrogen in various hydrogen productions, which usually involves the hydrogen evolution reaction (HER). Therefore, it is very important to explore a stable, low cost, economically and environmentally friendly and high efficiency HER catalyst for realizing large-scale industrial electrocatalytic hydrogen production [4]. 

Platinum and its alloys are considered to be the best active electrocatalyst to facilitate HER in acid condition because of its small overpotential and accelerating reaction rates [5], which can lead to more efficient water-splitting [6]. However, the high cost and low abundance of platinum limits its widespread application in practice [7,8]. Thus, it is highly necessary to explore earth-abundant and non-noble-metal HER electrocatalysts to substitute for platinum. Recently, many promising HER electrocatalysts have been unveiled, such as anatase [9], metal dioxides [10], carbides [11], selenides [12], sulfides [13], phosphides [14] and nitrides [15]. Among potential materials considered thus far, the family of transition metal phosphides (TMPs) has drawn a wide range of interests as a promising alternative catalyst for HER due to its high catalytic activities [16,17]. For instance, TMPs, including W-P [18,19], Fe-P [20,21], Ni-P [22,23] and Mo-P [24,25], are well known for their high catalytic activity and durability. Experimentally, many approaches were used to synthesize TMPs, such as the solvothermal method [26], hypophosphite thermal decomposition [27,28], high vacuum solid-state reaction method [28], metal-organic framework phosphatized method [29] and so on. Recently, researchers found TMPs with higher phosphorous component holds a higher catalytic activity for HER [29,30,31]. 

As a typical TMP with higher phosphorous component, great stability and good electronical conductivity,${\;\mathrm{MoP}}_{2}$ has attracted tremendous attention from academia [32,33,34]. Experimentally, a facile two-step strategy was used to fabricate ${\mathrm{MoP}}_{2}$ nanoparticle films on a metal Mo plate showing superior HER catalytic activity at all pH values [35]. Through a phosphidation reaction based on an ${\mathrm{MoS}}_{2}$ nanosheet array on carbon cloth, an ${\mathrm{MoP}}_{2}$ nanosheet was fabricated by Zhu et al. [36]. They found that the ${\mathrm{MoP}}_{2}$ nanosheet delivers superior catalytic activity and stability with an overpotential as low as 58 mV to drive a catalytic current density of 10 mA cm^−2^. In addition, Wu et al. [28] synthesized ${\mathrm{MoP}}_{2}$ nanoparticles with a high vacuum solid-state reaction method and carried out electrochemical tests. It was revealed ${\mathrm{MoP}}_{2}$ nanoparticles show superior HER performance with small overpotentials of 38 mV and low Tafel slopes of 52 mV dev^−1^. More recently, Gao et al. [37] synthesized ${\mathrm{MoP}}_{2}$ nanosheets using ${a―\mathrm{MaO}}_{3}$ nanosheets as a precursor by high temperature solid state reaction with phosphorus under vacuum conditions. It was found that ${\mathrm{MoP}}_{2}$ nanosheets exhibit very promising HER activity, considerably better than bulk and non-exfoliated ${\mathrm{MoP}}_{2}$. To our best knowledge, though there are many exciting experimental findings, insights into ${\mathrm{MoP}}_{2}$ for the systematical mechanism of hydrogen absorptions are lacking, which is of critical importance for further enhancing the activities and stabilities of HER electrocatalysts.

Gibbs free energy of hydrogen adsorption $\left(\Delta G_{H}\right)$ is usually used as a descriptor to predict HER activity of the electrocatalyst [38]. Nørskov et al. [39,40] reported that the maximum of HER activity is obtainable with the value of $\Delta G_{H}$ closing to zero. More negative values of $\Delta G_{H}$ mean the binding between hydrogen atom and facet is stronger, which is impedimentary for desorption of $H_{2}$. On the contrary, more positive values of $\Delta G_{H}$ stand for the weaker blinding between hydrogen atom and facet. The step of proton/electron-transfer can be hindered with the positive value. Therefore, the best-performing and ideal electrocatalyst for HER should have a value of $\Delta G_{H\;}$ close to zero. 

To figure out HER mechanisms of ${\mathrm{MoP}}_{2}$, the adsorption structures and energetics of atomic hydrogen have been systematically calculated using periodic density functional theory (DFT). HER activities of the surface were predicted by the value of $\Delta G_{H}$. Ab initio atomistic thermodynamics was employed to determine the most stable phase at 1 atm $H_{2}$ pressure and 298 K. Bader analysis [41] and different charge density [42] analysis were employed to further understand the interaction between Mo and P atoms. Finally, the doping effects on HER activity were investigated by substituting P with an S or N atom, as well as Mo atom with an Fe, Cu, Cr, Co, Mn or Ni atom, respectively. We hope our results can shed new light on further understanding of HER for and designing transition phosphide HER electrocatalysts.

## 2. Computational Methods

The spin-polarized periodic DFT calculations were performed by the plane-wave basis set with the projector augmented plan wave (PAW) method for treating core and valence electronics [43,44]. Perdew-Burke-Ernzerh (PBE) functional with generalized gradient approximation (GGA) [45] was carried to deal with the electronic exchange-correlation interaction. All calculations were implemented in the Vienna Ab initio Simulation Package (VASP) [46]. The plane-wave cutoff energy was tested using a well-known variable-controlling approach and set to 400 eV. The Gaussian smearing method was employed to represent the total energy with a smearing width of 0.02 eV. The low Miller-index facets were modeled by cleaved with the optimized structure of bulk MoP_2_, with a four-atom-layer 1 × 2 × 1 supercell for (100) facet and four-atom-layer 2 × 1 × 1 supercells for (111), (110), (101), (001) and (011) facets. To avoid the interaction among the adjacent images, 15 Å of vacuum along the perpendicular catalyst facets was added. The Monkhorst-Pack method with a centered k-point mesh of 3 × 3 × 1 was used to sample the Brillouin zone. The top half of the layers together with the added hydrogen atoms was relaxed during the geometry optimization in the whole calculations. During the structure optimization, the residual forces have converged to less than 0.025eV Å^−1^ and the total energy converged to less than 1.0×10^−6^ eV. To obtain a deep understanding of HER, the partial atomic charges were investigated using Bader charge analysis developed by Henkelman et al. [41]

The surface energies are determined as:

(1) $$\gamma \;=\;\frac{E_{s\mathrm{lab}}-NE_{\mathrm{bul}k}}{2A}$$

where $\;E_{\mathrm{slab}}$ and $\;E_{\mathrm{bulk}}$ are the total energy of the surface slab and the bulk, respectively. *N* is the number of formula unit in the slab and *A* is the facet area of the optimized slab. The differential hydrogen adsorption energy $\;\Delta E_{H}\;$ is defined as:

(2) $$\Delta E_{H\;}=E\left({\mathrm{MoP}}_{2}+\mathrm{nH}\right)-E\left[{\mathrm{MoP}}_{2}+\left(n-1\right)H\right]-\frac{1}{2}E\left(H_{2}\right)$$

where $E\left({\mathrm{MoP}}_{2}+\mathrm{nH}\right)\;$ and $E\left[{\mathrm{MoP}}_{2}+\left(n-1\right)H\right]$ are the total energy of the ${\mathrm{MoP}}_{2}$ system with n and (n-1) hydrogen atoms adsorbed on the surface. $E(H_{2})$ is the total energy of $H_{2}$ molecule in the gas phase. A negative value of $\;\Delta E_{H}$ suggests beneficial absorption. The differential Gibbs free energy of the adsorbed hydrogen atom is calculated by:

(3) $$\Delta G_{H}=\Delta E_{H}-T\Delta S_{H}+\Delta E_{\mathrm{ZPE}}$$

here, $\Delta E_{H}$ is the adsorption energy of the hydrogen atom determined by Equation (2). $\Delta E_{\mathrm{ZPE}}$ and $\Delta S_{H}$ stand for the zero-point energy correction and entropy change between adsorbed hydrogen and hydrogen under standard conditions at the temperature of T.$\;\Delta S_{H}$ can be obtained by $\;\Delta S_{H}≅-\frac{1}{2}S_{H_{2}}^{0}$; here, $S_{H_{2}}^{0}$ is the entropy of an isolated hydrogen molecular in gas phase at standard condition. The value of $\;T\Delta S_{H}$ is approximately equal to −0.2 eV. $\Delta E_{\mathrm{ZPE}}\;$ is defined as:

(4) $$\Delta E_{\mathrm{ZPE}}=E_{\mathrm{ZPE}}^{\mathrm{nH}}-E_{\mathrm{ZPE}}^{\left(n-1\right)H}-\frac{1}{2}E_{\mathrm{ZPE}}^{H_{2}}$$

where $E_{\mathrm{ZPE}}^{\mathrm{nH}}$, $E_{\mathrm{ZPE}}^{\left(n-1\right)H}$ and $E_{\mathrm{ZPE}}^{H_{2}}$ are the zero-point energy of n and (n-1) hydrogen atoms adsorbed on the surface, as well as a gas phase hydrogen molecular, respectively. The $E_{\mathrm{ZPE}}^{H_{2}}$ represents the zero-point energy of hydrogen molecule in gas phase. The zero-point energy can be calculated by:

(5) $$E_{\mathrm{ZPE}}=\overset{i}{\underset{1}{\sum }}\frac{1}{2}hv_{i}$$

where *h* represents the Plank constant and v is the vibrational frequency. The calculated value of the vibrational frequency is 4301 cm^−1^ for a single hydrogen molecular, which is consistent with the experimental finding of 4395 cm^−1^ [47]. The calculated values of vibrational frequencies are 1744.53 cm^−1^, 608.71 cm^−1^ and 548.19 cm^−1^ for hydrogen adsorbed on the (111) facet. Using Equation (4) and Equation (5), the values of 0.04 eV for $\Delta E_{\mathrm{ZPE}}$ was obtained for the (111) facet. Thus, the expression of $\Delta G_{H}$ can be represented as $\Delta G_{H}=\Delta E_{H}+0.24$ for the (111) facet.

To further identity the most stable phase, the Ab initio atomistic thermodynamics [48] was employed to determine $\Delta G_{H}$ at a specific temperature and pressure denoted by $\Delta G^{\mathrm{ad}}\left(T,p\right)$, which can be obtained by:

(6) $$\Delta G^{\mathrm{ad}}\left(T,p\right)=\frac{1}{A}\left[E^{\mathrm{total}}\left(N_{H}\right)-E^{\mathrm{total}}\left(0\right)-\frac{N_{H}}{2}E_{H_{2}}^{\mathrm{total}}-N_{H}\Delta \mu_{H}\left(T,p\right)\right]$$

where $\;E^{\mathrm{total}}\left(N_{H}\right)\;$ is the total energy of the system with the $N_{H}$ adsorbed hydrogen atoms on the surface, $E^{\mathrm{total}}\left(0\right)$ is the total energy of the clean facet, $E_{H_{2}}^{\mathrm{total}}$ is the total energy of $H_{2}$ molecule in gas phase and $\Delta \mu_{H}\left(T,p\right)$ is the hydrogen chemical potential at a given temperature and pressure. Clearly, $\Delta G^{\mathrm{ad}}\left(T,p\right)$ is a function of $\Delta \mu_{H}\left(T,p\right)$ for a surface at a concrete hydrogen coverage. $\Delta \mu_{H}\left(T,p\right)$ based on the specific condition can be determined by:

(7) $$\Delta \mu_{H}\left(T,p\right)=\Delta \mu_{H}\left(T,p^{\theta }\right)+\frac{1}{2}k_{B}T\ln\left(\frac{P}{P^{\theta }}\right)$$

here, $\Delta \mu_{H}\left(T,p^{\theta }\right)$ is the hydrogen chemical potential at the standard pressure $p^{\theta }$ and $k_{B}$ is Boltzmann constant.

To investigate the kinetic mechanism of HER, the activation energies were calculated based on the transition theory. The activation energy barrier ($E_{a}$) and reaction energy (ΔE) of the elemental reaction are obtained by the following formulas:

(8) $$E_{a}=E_{TS}-E_{IS}$$

(9) $$\Delta E=\;E_{FS}-E_{IS}$$

where $E_{\mathrm{IS}}$, $E_{\mathrm{TS}}$ and $E_{\mathrm{FS}}$ represent the total energy of initial state, transition state and final state, respectively. 

## 3. Results and Discussion

### 3.1. Electronic Structures of Bulk ${\mathrm{MoP}}_{2}$

**Figure 1:**
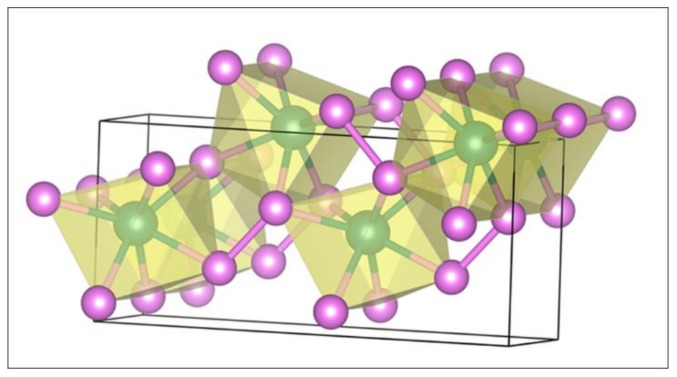
Bulk ${\mathrm{MoP}}_{2}$. Mo: dark green; P: purple.

Figure 1 shows the bulk ${\mathrm{MoP}}_{2}$, which has an orthorhombic structure with a space group of Cmc21 (36). A Mo atom is seven-coordinated by six P atoms at ends of the prism and one P atom at outside of the rectangular facet of the prism, which shows five ring-pore structure and the zigzagging layered with a relatively large free volume. Table 1 lists the optimized lattice parameters that are in good agreement with the available experimental findings [28,49] and recent theoretical results [50]. The maximum error is less than 0.06 Å between the calculated and experimental findings. 

In surface science studies, priority will be given to low Miller-index facets due to their high durability. For orthorhombic ${\mathrm{MoP}}_{2}$, the low Miller-index facets can be cleaved to (100), (110), (111), (011), (101) and (001) examples. The optimized structures of these facets are plotted in Figure S1 and corresponding surface energies are summarized in Table 2. Generally, the most stable facet has the lowest surface energy and can be synthesized in experiments [51,52,53,54]. From computing surface energy, it can be found the thermodynamic stability of the clean facets follows the order of (111) > (110) > (101) > (011) > (100) > (001). Thus, the most stable facet is (111), which agrees with the recent experimental finding where the (111) facet has been synthesized [54]. In addition, surface packing densities (SPD) are usually used to identify the stability. A larger value of SPD means that it is more stable. Table 2 also lists the calculated data of SPD for each facet. Likewise, the (111) facet is recognized as the most stable one. Therefore, the (111) facet is further examined. 

**Figure 2:**
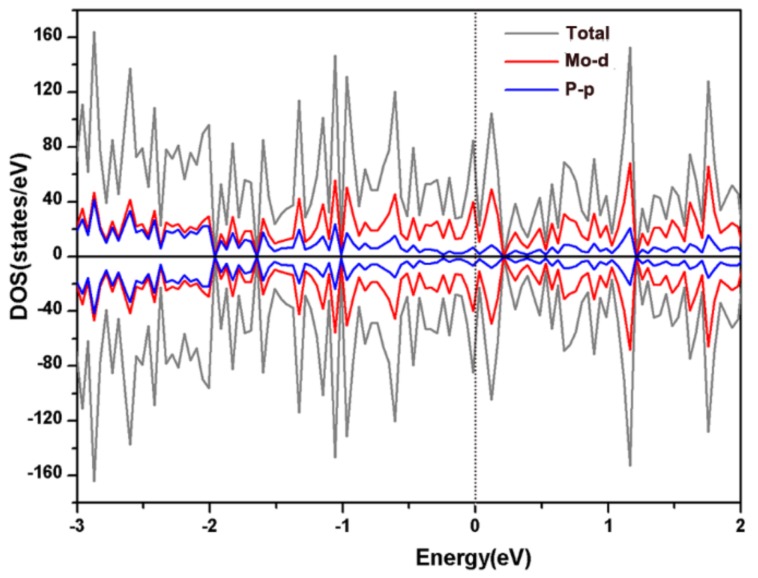
Project and total density of states of the (111) facet.

To further understand the electronic structure of the (111) facet, Figure 2 sketches the calculated total and project electronic density of states (TDOS and PDOS, respectively) data. Obviously, the facet shows metallic behavior with none-zero TDOS data at the Fermi level. It is advantageous for good conductivity which is beneficial for HER activity. The main contributions of TDOS are from Mo-d orbitals. 

### 3.2. Hydrogen Adsorption

**Figure 3:**
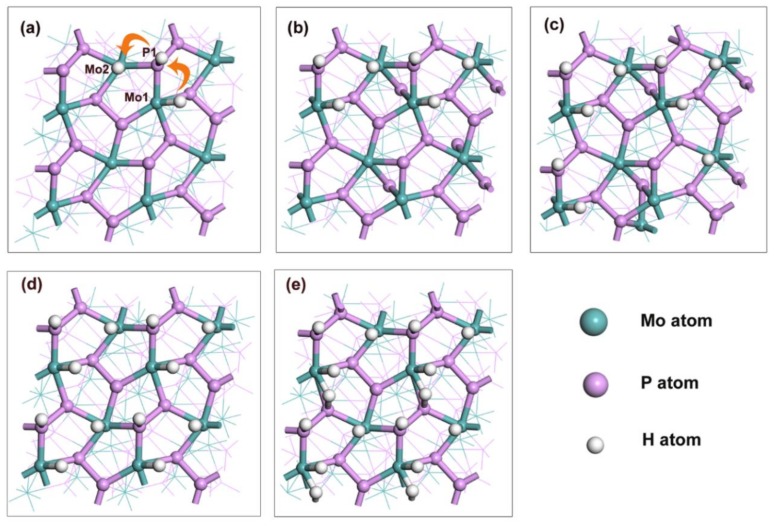
Optimized structures of the (111) facet with the H coverages: (**a**) 18.75%, (**b**) 37.5%, (**c**) 56.25%, (**d**) 75% and (**e**) 100%.

To investigate HER activity, all possible adsorption sites on the (111) facet were carefully tested. It was revealed that there are six favorable adsorption sites located at Mo1, P1, bridge (Mo1-P3), Mo2, bridge (Mo1-P2) and P2, as shown in Figure S2. The absorption energies at each site were listed in Table S1, which follows the trend: Mo1 < P1 < bridge (Mo1-P3) < Mo2 < bridge (Mo1-P2) < P2. With the assumption that hydrogen atoms easily spread over the facet at the lowest energy sites for each type of hydrogen coverage, we depicted the global minimum of hydrogen adsorption for each type of hydrogen coverage on the (111) facet. Figure 3 shows the optimized structures with different hydrogen coverages (here, 100% coverage means adsorbed 16 H atoms on the (111) facet). It is usually thought hydrogen coverage should first occupy all sites with the lowest adsorption energies, i.e., all Mo1 sites on the (111) facet. Strangely, when the first H atom takes over one Mo1 site on the (111) facet, the second H atom do not go to the other Mo1 site. It occupies one P1 site and the third H atom occupies an adjacent Mo2 site, as shown in Figure 3a. Subsequently, occupancy sites of H atoms will repeat the procedure until reaching 75% coverage. For the last 75–100% coverage, additional H atoms prefer to adsorb on the bridge (Mo1-P3). Interestingly, there are no H atoms adsorbed on bridge (Mo1-P2) and P2 sites during the processes of hydrogen coverage. To further understand these phenomena, the adoption energies and charge density differences were investigated.

**Figure 4:**
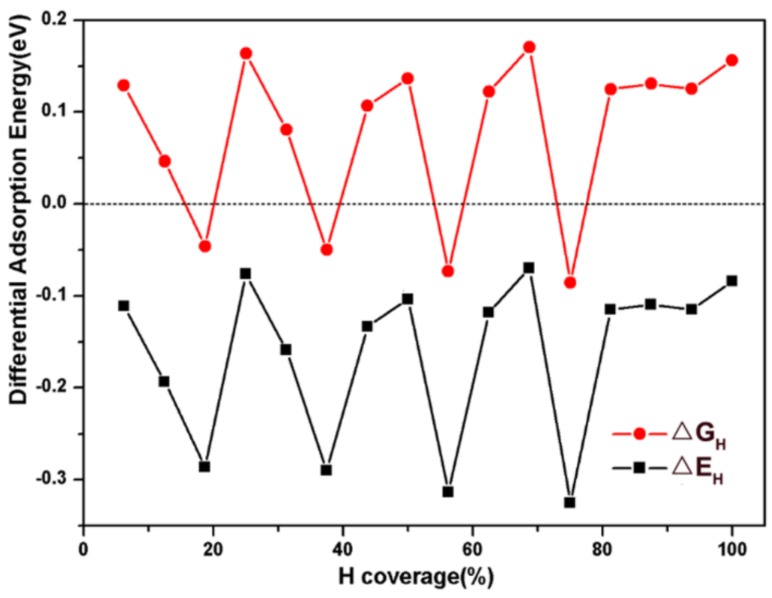
Adsorption energy ($\Delta E_{H}$) and adsorption free energy ($\Delta G_{H}$ ) as functions of hydrogen coverage on ${\mathrm{MoP}}_{2}$.

Figure 4 describes the calculated values of $\Delta E_{H}$ and $\Delta G_{H}$ for H adsorption on the (111) facet with different hydrogen coverages. The absolute values of $\Delta G_{H}$ are less than 0.2 eV, which is a favorable value for HER. Therefore, it is indicated that the (111) facet can favor HER over a large hydrogen coverage range. The zigzagged patterns before 75% hydrogen coverage suggest a synergy among Mo1, P1 and Mo2 sites, which are attributed to repeating occupancy sites of H atoms. 

To obtain a deep understanding, herein, the charge density difference induced by the adsorbed H atoms on the (111) facet were analyzed. The charge density differences for H atoms adsorbed on the surface are defined by $\Delta \rho =\rho^{\mathrm{sys}}-\rho^{\mathrm{sur}}-\rho^{H}$, where $\rho^{sys}$, $\rho^{sur}$ and $\;\rho^{H}$ are the charge densities of the catalytic surface adsorbed H atoms system, the clean surface and H atoms, respectively. 

**Figure 5:**
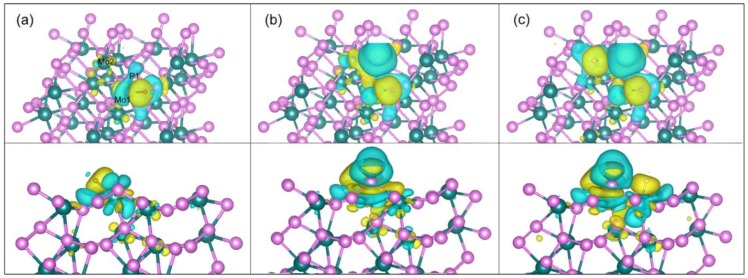
The charge density differences for the adsorbed H atoms with H coverage of (**a**) 1/16, (**b**) 2/16 and (**c**) 3/16. Top and bottom panels represent overlooking and sidelooking, respectively. Color codes for Mo, P and H are dark green, purple and brown, respectively. Charge accumulation and depletion are plotted by the yellow and light blue regions with the isosurface value of 1.5×10^−3^ e/bohr^3^.

Figure 5 shows the charge density differences for the system made up of the (111) facet and the adsorbed H atoms with H coverage of 1/16, 2/16 and 3/16. For 1/16 H coverage at Mo1 site, there are significant electron accumulation (yellow) and depletion (light blue) around the adsorbed H atom and the connected Mo atom, respectively. The election transfer mainly exists between H and Mo atoms, indicating a strong interaction between them. However, no electron depletion is found around the H atom, revealing that many electrons transferred from the Mo to the H atom. Interesting, a small amount of the electron depletion and accumulation are found around Mo1-P1 and P1-Mo2 bonds, respectively, which predict that the bond length of Mo1-P1 will increase, while that of P1-Mo2 will decrease compared with those in a clean facet, agreeing with the analysis of bond lengths from the geometry optimization in Table 3. Meanwhile, the small electron depletion of P atom at P1 site and accumulation of Mo atom at Mo2 site are in line with that from the Bader analysis in Table 3. Small amounts of charge density differences on the P1 site due to the occupation of the Mo1 site will affect the adsorption energy on the P1 site compared with that of only H coverage at the P1 site in Table 4, in which the adsorption energy will decrease. In other words, H adsorption at Mo1 site facilitates hydrogen adsorption at the P1 site, agreeing with a zigzag pattern with H coverage discussed above. For 2/16 H coverage, where the adsorbed H atoms are at both Mo1 and P1 sites, an accumulation (yellow) forms between H and P atoms due to a bonding generated between them. In addition, a depletion of the electron density outside the adsorbed H atom connected P atom occurs because of the rearrangement of electron toward the bond of H and P. When H coverage increase to 3/16, the phenomenon of electron accumulation and depletion around the H atom and connected Mo atom at Mo2 site are analogous to that of 1/16 H coverage. 

Overall, H adsorption will lead to a rearrangement of electrons and change the bond length of Mo-P on the (111) facet shown in Table 3. Meanwhile, H adsorption at one site will facilitate hydrogen adsorption at the other site shown in Table 4. 

**Figure 6:**
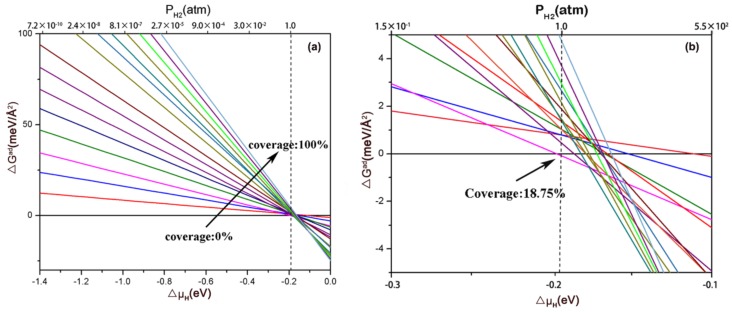
$\Delta G^{\mathrm{ad}}$ at 298K with pressure and $\Delta \mu_{H}\;$ ranging from: (**a**) $\Delta \mu_{H}\;$ −1.4 to 0 eV and (**b**) −0.3 to −0.1 eV. Note that the dot line is used to clearly reflect the values of $\Delta G^{\mathrm{ad}}\left(T,p\right)$ at 1 atm $H_{2}$ and 298 K. The intersection points show the values of $\Delta G^{\mathrm{ad}}\left(T,p\right)$ for different coverages at 1 atm $H_{2}$ and 298K.

Furthermore, to estimate the most stable coverage of H on the (111) facet at 1 atm $H_{2}$ and 298K, ab initio atomistic thermodynamics was employed to identify the $\Delta G^{\mathrm{ad}}\left(T,p\right)\;$ by the hydrogen chemical potential $\Delta \mu_{H}$ and pressure illustrated in Figure 6. Each line represents a given H coverage. Obviously, the most stable phase at 1 atm $H_{2}$ and 298 K is 18.75% H coverage, in which the active sites for HER is the Mo2 site. As shown in the Figure S3, the kinetic energy barrier of the Tafel pathway was considered by calculating the kinetic energy barrier profiles of the formation of Hydrogen, from which the HER activity of electrocatalysts can be known. The kinetic energy barrier profiles for the Tafel path at 18.75% H coverage is 0.75 eV, which is lower than the previous result of 1T-${\mathrm{MoS}}_{2\;}$ (0.85 eV) [41]. Based on the exiting learning, it can be learned that the lower energy barrier may facilitate the formation of the H-H bond. 

To understand the kinetic mechanism of HER at 18.75% H coverage, the activation energies of the Tafel reaction were calculated, in which two adjacent pre-bonded H atoms at top site of Mo2 and P1 combine to form an $H_{2}$ molecule. Figure S3 shows the kinetic energy barrier profile of HER on the (111) surface at 18.75% H coverage. Clearly, $H_{2\;}$ generation needs to experience two transition states. The first step needs to overcome a relatively high activation energy barrier of 0.75 eV. However, it drops to 0.18 eV for the second step. These low activation energy barriers revel that $H_{2\;}$ generation through Tafel reaction is favored on the (111) surface at 18.75% H coverage. 

### 3.3. Doping on the ${\mathrm{MoP}}_{2\;}$(111) Surface

Doping has been extensively used to tune the physical and chemical properties of materials. It has been proven that doping on transition-mental is an effective way, which influence the chemical activity of catalysts [55,56]. The hydrogen generation of catalytic activity of 2H ${\mathrm{MoS}}_{2\;}$ can be promoted by the introduction of metal dopants such as Fe, Ni and Co [57,58,59]. Experiments found that N, S doping of MoP can enhance the HER activity [60,61]. To systematically recognize HER activity of ${\mathrm{MoP}}_{2\;}$ the doping effects on HER activity for the (111) facet of ${\mathrm{MoP}}_{2\;}$ have been done from a theoretical perspective.

**Figure 7:**
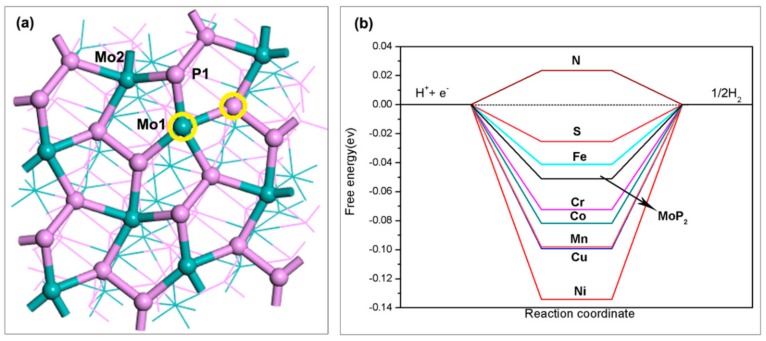
(**a**) Top view of the (111) facet with two doping atom sites. (**b**) Gibbs free energies of adsorption for the (111) facet and its doped facets.

Figure 7a illustrates the substitutional doing of the Mo site with a Co, Cu, Mn, Ni, Fe or Cr atom, and doping of the P site with an S or N atom was evaluated. The Hubbard correction was considered for the metal dopant of Fe. Geometry optimizations of the doping system show that doping of the Mo site by using a Co, Fe, Mn, Ni or Cr atom, as well as doping of the P site by using a N or S atom could not induce visible structural change to the (111) facet. On the other hand, doping with a Cu atom induces slightly larger lattice distortion, which is 0.8% tensile strain, compared with the clear (111) facet. The calculated $\Delta G_{H}$ of the doped system was summarized in Figure 7b. Clearly, doping of the P site with an N or S atom and doping of the Mo site with an Fe atom give a value of $\Delta G_{H}$ closer to zero, which promote HER catalytic activity for the (111) facet of MoP_2_.

## 4. Conclusions

In this work, HER catalytic activities for the (111) facet of ${\mathrm{MoP}}_{2\;}$ were systematically investigated from theoretical perspective using periodic DFT calculations. The electronic structure, Gibbs free energy of hydrogen adsorption and ab initio thermodynamics of hydrogen coverage were calculated to reveal the HER characteristics of ${\mathrm{MoP}}_{2\;}$.

From the electronic structures of six low Miller-index facets, we found the thermodynamic stability of the clean facets follows the order of (111) > (110) > (101) > (011) > (100) > (001). As the most stable facet, the (111) facet has the metallic behavior with none having zero DOS at the Fermi level. The values of $\Delta G_{H}$ indicated that the (111) facet can favor HER over a large hydrogen coverage range. The zigzagged patterns before 75% hydrogen coverage suggest a synergy among Mo1, P1 and Mo2 sites, which are attributed to repeat occupancy sites of H atoms. From charge density differences and Bader analysis, we found that H adsorption will lead to a rearrangement of the electron and change the bond length of Mo-P on the (111) facet. Meanwhile, H adsorption at one site will facilitate hydrogen adsorption at the other site. From the ab initio atomistic thermodynamics analysis, we obtained that the most stable phase at 1 atm $H_{2\;}$ and 298 K is 18.75% H coverage, in which the active sites for HER is Mo2 site. Finally, we found doping of the P site with an N or S atom and doping the Mo site with an Fe atom can promote HER catalytic activity for the (111) facet of ${\mathrm{MoP}}_{2\;}$.

## Figures and Tables

**Table 1:** The lattice parameters of bulk ${\mathrm{MoP}}_{2}$_._

$\mathrm{Bulk}\;{\mathrm{MoP}}_{2}.$	a (Å)	b (Å)	c (Å)
Present	3.147	11.242	5.009
Calculation ^a^	3.142	11.132	4.949
Experiment ^b^	3.145 ^a^	11.184	4.984

^a^. Reference [50] from density functional theory (DFT) with Perdew-Burke-Ernzerh (PBE) functional. ^b^. Reference [49] from experimental data by X-Ray power method.

**Table 2:** Calculated surface energies and surface packing density (SPD) of low Miller-index facets for ${\mathrm{MoP}}_{2}$_._

${\mathrm{MoP}}_{2}\;\mathrm{Facet}$	(111)	(110)	(101)	(011)	(100)	(001)
Surface energy(meVÅ^−2^)	104.78	134.34	134.85	146.73	147.35	173.52
SPD (atom nm^−2^)	18	17	15	13	11	11

**Table 3:** Bond lengths and partial atomic charges from Bader analysis with H coverage of 1/16, 2/16 and 3/16.

	Clean facet	1/16 H atom	2/16 H atoms	3/16 H atoms
$r_{{\mathrm{Mo}}_{1}-P_{1}}$ (Å)	2.313	2.330	2.380	2.394
$r_{p_{1}-{\mathrm{Mo}}_{2}}$ (Å)	2.382	2.366	2.435	2.411
$P1_{Bader}$(e)	−0.360	−0.354	0.146	0.200
$Mo1_{Bader}$(e)	0.659	0.707	0.722	0.736
$Mo2_{Bader}$(e)	0.696	0.670	0.681	0.698

**Table 4:** Adsorption energies at the Mo1, P1 and Mo2 sites. Here, 1 means there is an adsorbed H atom, while 0 stands for 0 adsorbed H atoms.

Adsorption Energy	Mo1 site	P1 site	Mo2 site
$\Delta E_{H}\left(eV\right)$	−0.106	0	0
	0	−0.038	0
	0	0	0.083
	1	−0.194	0
	1	1	−0.286

## Supplementary Materials

The following are available online at https://www.mdpi.com/2079-4991/9/9/1270/s1, Figure S1: All possible adsorption sites on (111) facet, Figure S2. Top view of six clean MoP2 surfaces, including (a) (100), (b) (111), (c) (110), (d) (001), (e) (101) and (f) (011) facets. Mo: dark green; P: purple. Table S1. Adsorption energy of the optimized hydrogen adsorption sites for (111) facet.
