# Supplementary material for: New Insight on Hydrogen Evolution Reaction Activity of MoP2 from Theoretical Perspective
Source: Nanomaterials (Basel). 2019 Sep 5;9(9):1270. doi: 10.3390/nano9091270 (PMC6781081; doi:10.3390/nano9091270)
Supplement: Supplementary file 1 [file nanomaterials-09-01270-s001.pdf]

Support information

New Insight on Hydrogen Evolution Reaction Activity of MoP_2 from Theoretical Perspective

Yuyue Gao [1,2], Hongyan Li [1,2], Jingyu Wang [3], Jianyi Ma [1] and Haisheng Ren [2,*]

1 Institute of Atomic and Molecular Physics, Sichuan University, Chengdu, 610065, China; jianyima@scu.edu.cn
2 School of Chemical Engineering, Sichuan University, Chengdu, 610065, China;
3 School of Aeronautics and Astronautics, Sichuan University, Chengdu, Sichuan 610065, China; wangjingyu@scu.edu.cn
* Correspondence: renhs@scu.edu.cn; Tel./Fax: +86 2885402951

Table S1. Adsorption energy of the optimized hydrogen adsorption sites for (111) facet. (unit in eV)

Adsorption sites	Mo1	P1	Mo2	P2	Mo1-P2	Mo1-P3
111-facet	-0.106	-0.038	0.083	0.402	0.251	0.016

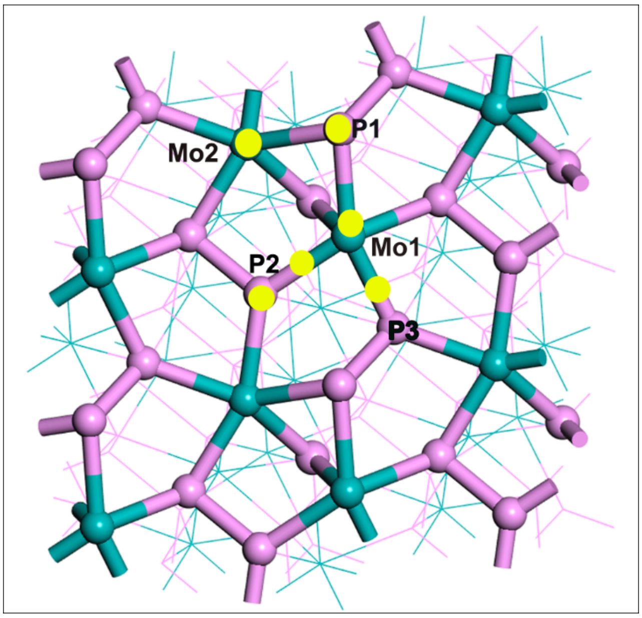

Figure S1. All possible adsorption sites on (111) facet.

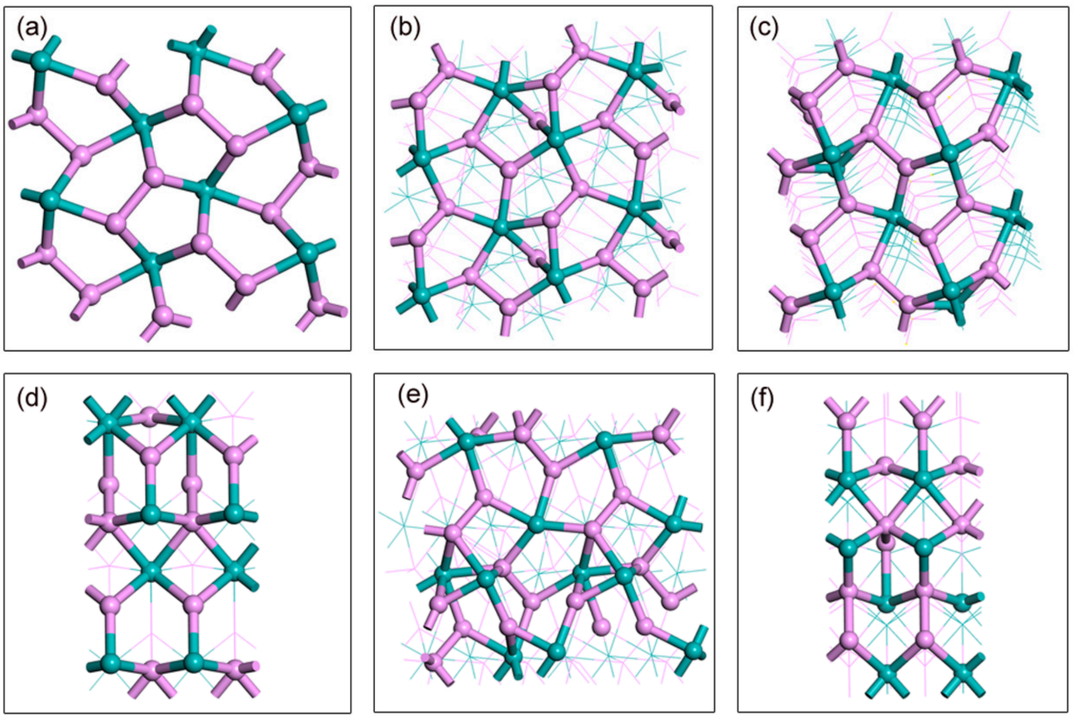

Figure S2. Top view of six clean MoP_2 surfaces, including (a) (100), (b) (111), (c) (110), (d) (001), (e) (101) and (f) (011) facets. Mo: dark green; P: purple.
